# Brain death and potential organ donors in neurocritical care mortality

**DOI:** 10.1186/2197-425X-3-S1-A897

**Published:** 2015-10-01

**Authors:** V Spatenkova, O Bradac, P Suchomel

**Affiliations:** Neurocenter, Neurointensive Care Unit, Regional Hospital, Liberec, Czech Republic; Military University Hospital and First Medical School, Department of Neurosurgery, Charles University, Prague, Czech Republic; Department of Neurosurgery, Neurocenter, Liberec, Czech Republic

## Introduction

Neurocritical care mortality has potential for organ donation due to brain death.

## Objectives

The aim of this study was to analyse the neurocritical care mortality rate and potential brain-dead organ donors.

## Methods

We performed an analysis of a 10-year prospective observational cohort database of 6138 patients (58.2% of males, mean: age 55.9 ± 14.7 years, body weight 78.3 ± 15.6 kg, body mass index 26.9 ± 4.7, NICU stay 3.8 ± 5.3 days, Acute Physiology and Chronic Health Evaluation II score on admission 10.63 ± 5.2) admitted to a single adult neurointensive care unit (NICU). There were 3462 (56.4%) patients (pts) with brain disease (stroke 43.2%, tumour 31.1%, trauma 13.6%, epilepsy 3.8%, hydrocephalus 3.4%, infection 2.5%, others 2.2%), 10.3% of pts had internal carotid artery stenosis (ACI), 32.6% of pts had spine diseases and 0.7% of pts had other disorders. Mean Glasgow Coma Scale on admission was 13.79 ± 2.51 and Glasgow Outcome scale upon discharge from NICU 3.97 ± 1.13.

## Results

From 6138 admitted patients there were 159 (2.6%) cases of NICU mortality with mean length of stay 9.21 ± 10.2. We found no differences in gender (p = 0.804), but mortality rate was significantly higher in acute admissions (p < 0.001), primary admissions and secondary to 24 hours than secondary after 24 hours (p < 0.001). Comparing the diagnoses, there was a significantly higher mortality rate in pts with brain diseases (95.6% of deceased pts, p < 0.001) than in ACI (0.6%), spine (1.9%) and from others (1.9%). From brain diseases there was significantly higher mortality in stroke pts (67.1%) than in trauma (17.8%), tumour (10.5%), hydrocephalus (2%), infection (2%) and epilepsy (0.7%), There were 23 (14.5%) pts with clinical signs of brain death, of which 13 (56.5%) became organ donors. Main reason of non-harvesting donors was hemodynamic instability (16.7%) and family reluctance (12.5%).

## Conclusions

The results of our prospective databases showed that brain damage is the most common cause of mortality in neurointensive care; however there was a low proportion of clinical sign of brain death and not all potential donors were harvested.

